# Factors influencing circuit lifetime in paediatric continuous kidney replacement therapies – results from the EurAKId registry

**DOI:** 10.1007/s00467-024-06459-6

**Published:** 2024-07-18

**Authors:** Anna Deja, Isabella Guzzo, Andrea Cappoli, Raffaella Labbadia, Aysun Karabay Bayazit, Dincer Yildizdas, Claus Peter Schmitt, Marcin Tkaczyk, Mirjana Cvetkovic, Mirjana Kostic, Wesley Hayes, Rukshana Shroff, Augustina Jankauskiene, Ernestas Virsilas, Germana Longo, Enrico Vidal, Sevgi Mir, Ipek Kaplan Bulut, Andrea Pasini, Fabio Paglialonga, Giovanni Montini, Ebru Yilmaz, Liane Correia Costa, Ana Teixeira, Franz Schaefer, for the ESCAPE Network

**Affiliations:** 1 Department of Paediatrics and Nephrology, Medical University of Warsaw, Warsaw, Poland; 2 Division of Nephrology, Dialysis and Transplant Unit, Bambino Gesù Children’s Hospital-IRCCS, Rome, Italy; 3 Department of Pediatric Nephrology, Cukurova University, Faculty of Medicine, Adana, Turkey; 4 Division of Pediatric Nephrology, Center for Pediatrics and Adolescent Medicine, Heidelberg University Hospital, Heidelberg, Germany; 5 Department of Pediatrics and Immunology, Nephrology Division, Polish Mothers Memorial Hospital Research Institute, Lodz, Poland; 6 Department of Nephrology, University Children Hospital, Belgrade, Serbia; 7 Department of Pediatric Nephrology, UCL Great Ormond Street Hospital and Institute of Child Health, London, UK; 8 Clinic of Pediatrics, Institute of Clinical Medicine, Vilnius University, Vilnius, Lithuania; 9 Pediatric Nephrology, Dialysis and Transplant Unit, Department of Woman’s and Children’s Health, University of Padua, Padua, Italy; 10 Department of Pediatric Nephrology, Ege University Faculty of Medicine, Izmir, Turkey; 11 Pediatric Nephrology and Dialysis, Pediatric Unit, IRCCS Azienda Ospedaliero-Universitaria Di Bologna, Bologna, Italy; 12 Pediatric Nephrology, Dialysis and Transplant Unit, Fondazione Ca’ Grande IRRCS, Ospedale Maggiore Policlinico, Milan, Italy; 13 Department of Pediatric Nephrology, Dr Behcet Children Research and Education Hospital, Izmir, Turkey; 14 Department of Pediatric Nephrology, Centro Materno-Infantil Do Norte, Porto, Portugal

**Keywords:** Acute kidney injury, Continuous kidney replacement therapy, Circuit lifetime, Regional citrate anticoagulation

## Abstract

**Background:**

Continuous kidney replacement therapy (CKRT) has recently become the preferred kidney replacement modality for children with acute kidney injury (AKI). We hypothesise that CKRT technical parameters and treatment settings in addition to the clinical characteristics of patients may influence the circuit lifetime in children.

**Methods:**

The study involved children included in the EurAKId registry (NCT 02960867), who underwent CKRT treatment. We analysed patient characteristics and CKRT parameters. The primary end point was mean circuit lifetime (MCL). Secondary end points were number of elective circuit changes and occurrence of dialysis-related complications.

**Results:**

The analysis was composed of 247 children who underwent 37,562 h of CKRT (median 78, IQR 37–165 h per patient). A total of 1357 circuits were utilised (3, IQR 2–6 per patient). MCL was longer in regional citrate anticoagulation (RCA), compared to heparin (HA) and no anticoagulation (NA) (42, IQR 32-58 h; 24, IQR 14-34 h; 18, IQR 12-24 h, respectively, p < 0.001). RCA was associated with longer MCL regardless of the patient’s age or dialyser surface. In multivariate analysis, MCL correlated with dialyser surface area (beta = 0.14, p = 0.016), left internal jugular vein vascular access site (beta = -0.37, p = 0.027), and the use of HA (beta = -0.14, p = 0.038) or NA (beta = -0.37, p < 0.001) vs. RCA. RCA was associated with the highest ratio of elective circuit changes and the lowest incidence of complications.

**Conclusion:**

Anticoagulation modality, dialyser surface, and vascular access site influence MCL. RCA should be considered when choosing first-line anticoagulation for CKRT in children. Further efforts should focus on developing guidelines and clinical practice recommendations for paediatric CKRT.

**Graphical abstract:**

**Figure:**
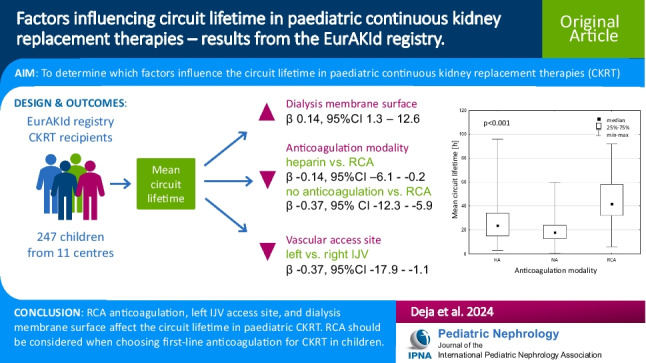
A higher resolution version of the Graphical abstract is available as Supplementary information

**Supplementary Information:**

The online version contains supplementary material available at 10.1007/s00467-024-06459-6.

## Introduction

Acute kidney injury (AKI) is a common condition with an increasing incidence in the paediatric population [1]. A recent meta-analysis encompassing studies from 26 countries revealed that AKI was diagnosed in 26% of hospitalised children across high-, middle-, and low-income countries [2]. In a paediatric intensive care unit setting, a large prospective multinational study corroborated a comparable incidence of AKI, with severe AKI being observed in 11.6% of cases [3]. Furthermore, AKI is linked to heightened risks of both short- and long-term adverse outcomes, including prolonged hospitalisation, chronic kidney disease, and mortality [3, 4].

Continuous kidney replacement therapy (CKRT) has become the preferred treatment modality for children with dialysis-dependent AKI [5]. CKRT holds superior appeal in critically ill children as it allows smooth fluid removal, avoids the disequilibrium syndrome seen with intermittent haemodialysis (iHD) and provides better purification efficacy and more precise fluid balance than peritoneal dialysis (PD) [6]. Nevertheless, the effectiveness of CKRT depends on the ability to preserve a circuit until it reaches its target lifetime. Premature filter clotting causes disruption and reduces the attainable ultrafiltration and solute clearance, while concurrently increasing blood loss and hemodynamic instability, workload, and treatment costs [7]. In the context of long-term therapy, effective anticoagulation strategies are of great importance. Unfortunately, up to 27% of dialyzers clot prematurely [8], necessitating unexpected circuit changes. In the adult population, RCA was proven to prolong the circuit lifetime compared to HA [9], and it is currently considered standard of care [10]. In recent years, RCA has also been successfully used in children, resulting in the creation of simplified protocols [11]. However, the absence of standardised paediatric protocols and the wide range of patient age, body mass and treatment indications, make CKRT in the paediatric population currently more challenging than in adults**.** Hence, it is essential to identify the modifiable factors affecting circuit survival time in order to develop clinical practice recommendations for paediatric CKRT. This study aimed to determine these factors and to evaluate the management and technicalities of CKRT procedures in children across Europe.

## Materials and methods

### EurAKId Registry

The EurAKId Registry (ClinicalTrials.gov NCT 02960867) was launched in September 2016 by the European Study Consortium for Chronic Kidney Disorders Affecting Paediatric Patients (ESCAPE) Network. It is a prospective, multicentre, international, observational study that collects data on acute kidney replacement therapies (KRT) in children using a web-based case report form. To date, 14 European paediatric nephrology centres have participated in the study. All participating institutions have obtained approvals from the local institutional review boards.

The registry collects data on children aged 0–18 years, treated with KRT at hospital admission or during hospitalisation, both in and outside PICU, and includes different dialysis modalities (PD, iHD, or CKRT) according to the local standards of care. The indications for acute KRT include AKI, as well as other reasons: metabolic decompensation, acute respiratory distress, sepsis, and fluid overload (FO). Children with known pre-existing chronic kidney disease are excluded. Defining AKI diagnosis, AKI staging, and exclusion of chronic kidney disease are the responsibility of on-site investigators.

Data capture involves demographic and baseline data, clinical data at the time of PICU admission and at the onset of dialysis, technical and procedural data related to the dialysis modality, and data on outcomes. All definitions used were reported previously [11].

### Study population

**Fig. 1:**
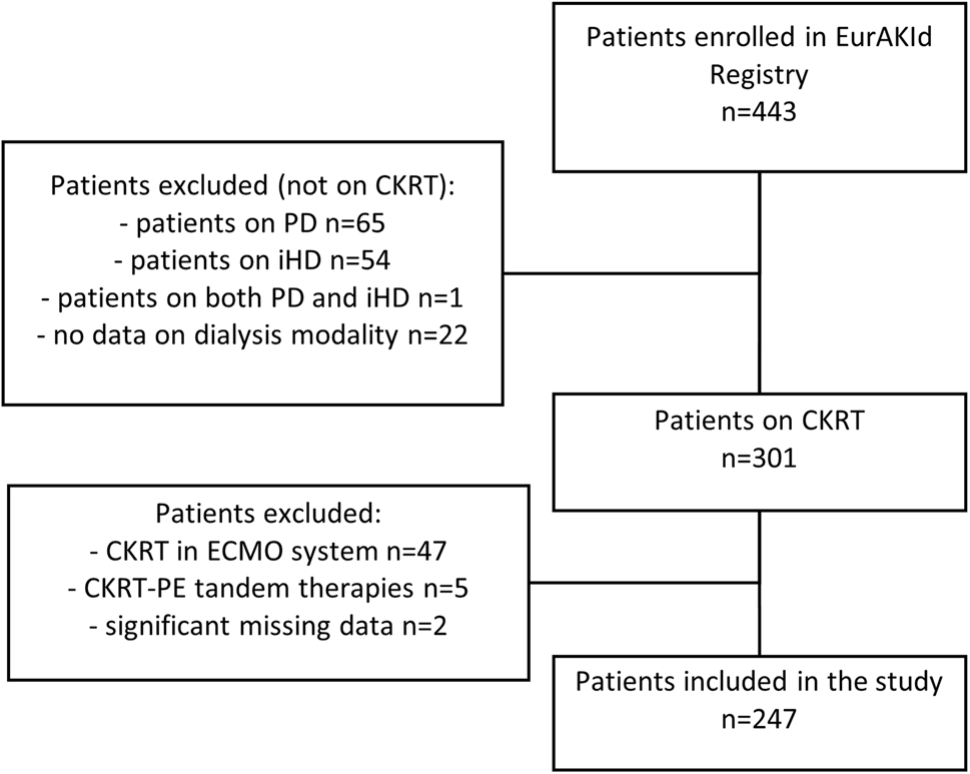
Patient selection

This study is a retrospective analysis of prospectively collected data focused on patients who received CKRT between September 2016 and September 2022. During this period, 443 patients were reported to the registry, of whom 301 from 11 centres received CKRT (Supplementary Fig. 1). After excluding 47 patients who underwent CKRT within an ECMO system, 5 children receiving tandem CKRT-plasma exchange, and 2 patients with significant missing data, 247 patients were included in the analysis (Fig. 1).

### Patient characteristics

From the data collected in the EurAKId Registry, we selected the baseline patient characteristics to define the examined population. The demographic and anthropometric data included: age [years], sex, race, initial body mass [kg], and BSA [m^2^]. Among the clinical parameters, we included the presence and stage of AKI, the presence of multi-organ dysfunction syndrome (MODS) and the number of organs involved, primary disease and comorbidities, the need for vasopressors use, the need for PICU admission, and fluid overload at the start of CKRT [%]. The primary disease was defined as the patient's principal disorder at the time of hospitalisation and comprised 12 categories: kidney disease, liver disease, pulmonary disease, cardiac disease, haematologic disease/bone marrow transplant (BMT), shock (including septic shock), malignancy/tumour lysis syndrome (TLS), inborn errors of metabolism (IEM), drug intoxication, immunologic disorder, crush syndrome, and neurologic disease. We included cardiac, pulmonary, neurologic, hepato-intestinal, hematologic, immunologic, septic, metabolic, and kidney comorbidities.

### Technical CKRT parameters and management

We analysed the technical aspects associated with CKRT initiation. These included the CKRT modality (CVVH, CVVHD, CVVHDF), vascular access site (right or left internal jugular vein, femoral vein, subclavian vein), dialysis membrane surface, and the administration and method of anticoagulation: no anticoagulation (NA), regional citrate anticoagulation (RCA), and heparin anticoagulation (HA). The dialysis filters were divided into three groups based on the dialysis membrane surface: small (membrane surface < 0.5m^2^), medium (between 0.5 and 1m^2^), and large (≥ 1m^2^). The analysed CKRT settings were: blood flow rate [ml/min], dialysate flow rate [ml/h], replacement fluid flow [ml/h], prescribed dialysis dose [ml/h], and ultrafiltration rate [ml/h]. All parameters were expressed as absolute values as well as adjusted for the patient’s body weight [ml/min/kg or ml/h/kg where applicable]. The prescribed dialysis dose was defined as the replacement flow rate (for CVVH), the dialysate flow rate (CVVHD), or the sum of both flow rates (for CVVHDF), and was expressed as absolute values as well as adjusted for the patient’s BSA [L/h/m^2^] and body weight [ml/h/kg]. We also assessed the total number of filters utilised and the total CKRT duration.

### Endpoints

The primary endpoint was the mean circuit lifetime expressed in hours, as reported in the EurAKId registry. The secondary endpoints were the number of elective circuit changes and the occurrence of dialysis-related complications. The filter change was considered elective in case of treatment termination due to diagnostic or therapeutic procedures, reaching the circuit target lifetime, and technical issues not associated with circuit malfunction. We defined four groups of complications: catheter dislocation, bleeding, thrombosis, and other complications.

### Statistical analysis

Collected data were statistically analysed using Dell Statistica 13.3 software.

The normality of data was assessed using the Shapiro–Wilk test. Depending on the distribution, data were expressed as mean ± standard deviation (SD) for variables with normal distribution or median (interquartile range — IQR) for variables with distribution other than normal. The following statistical tests were applied: Mann–Whitney U test for independent groups, Spearman’s rank correlation, Kruskal–Wallis ANOVA test, and chi-squared test. Multivariate analysis was performed using the general step-wise linear regression models. The variables were introduced into the model, excluding those correlated with each other with r > 0.60 to avoid collinearity. The criterion for inclusion in the final model was p < 0.050. The results of multivariate analyses were expressed as beta, confidence interval (CI), and p-value. The results were considered statistically significant, with p values < 0.050.

## Results

### Patient characteristics

**Fig. 2:**
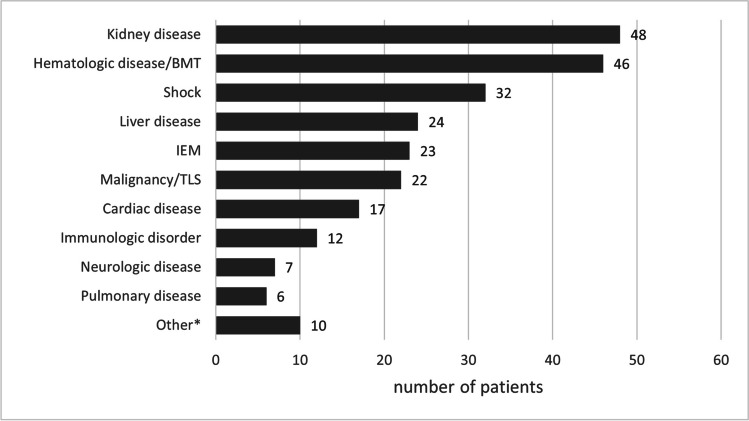
Primary disease occurrence in the examined population. *including drug intoxication (n = 3), crush syndrome (n = 3), no data (n = 4). BMT, bone marrow transplant; IEM, inborn errors of metabolism; TLS, tumor lysis syndrome

The general characteristics of the cohort are presented in Table 1. Median age at the start of CKRT was 4.1 (IQR 1.2–12) years. The study group comprised 23.1% of neonates and infants (up to 12 months of age, n = 57), 35.2% of young children (1–6 years, n = 87), 15.8% of children (6–12 years, n = 39), and 25.9% of adolescents (12–18 years, n = 64). The most common primary disease at hospital/PICU admission was kidney disease (19.4%, n = 48), followed closely by haematologic disease or BMT (18.5%, n = 46). The full distribution of primary diseases in the examined population is displayed in Fig. 2. The majority of patients had at least one comorbidity (79.4%, n = 196). Equal numbers of children were reported to have one, two, and three comorbidities (n = 64, 66, and 66, respectively).

**Table 1:** Demographic and clinical characteristics of the study group

Sex [n, boys/girls]	146/101
Age [years]	4.1 (1.2–12.0)
Weight at admission [kg]	16.0 (10.0–39.0)
BSA [m^2^]	0.67 (0.48–1.25)
Fluid overload [%]	4.0 (0.0–10.0)
Race [n, %]	
Caucasian	226 (91.5%)
Asian	16 (6.5%)
Black	2 (0.8%)
Unknown	3 (1.2%)
AKI [n, %]	195 (78.9%)
Stage	
1	12 (6.1%)
2	38 (19.5%)
3	141 (72.3%)
No data	4 (2.1%)
PICU admission [n, %]	216 (87.4%)
MODS [n,%]	151 (61.1%)
Number of organs involved [n, % of MODS]	
2	25 (16.6%)
3	68 (45.0%)
4	47 (31.1%)
> 4	11 (7.3%)
Vasopressor use [n, %]	145 (58.7%)

BSA, body surface area; AKI, acute kidney injury; PICU, pediatric intensive care unit; MODS, multiple organ dysfunction syndrome

Most of the patients required CKRT for AKI (78.9%, n = 195), mainly AKI stage 3 (72.3%, n = 141). Among the non-AKI patients, the three most common primary diseases were IEM (28.9%), followed by haematologic disease/BMT (21.1%), and liver disease (18.4%) (a full summary is available in Supplementary Fig. 2). Five of the non-AKI patients (11.2%) presented with fluid overload of 10% or more at CKRT initiation. There was no significant difference in fluid overload between the AKI and non-AKI patients (4.6%, IQR 0.0–11.6% vs 3.6%, IQR 0.0–8.7%, respectively, p = 0.157). Eighty-five per cent of CKRT procedures were conducted in the PICU setting. Thirty-one patients underwent another dialysis method during hospitalisation, either before or after CKRT (iHD 20 patients, PD 11 patients).

### CKRT technical parameters

Two hundred and forty-seven patients underwent a total of 37,562 h of CKRT, with a median time of 78 h (IQR 37–165) per patient. A total of 1357 circuits were utilised with a median of 3 (IQR 2–6) per patient. The majority of patients (n = 192, 77.7%) underwent CKRT on Prismaflex/Prismax devices, Aquarius was used in nine (3.6%), and CARPEDIEM (Cardio-Renal Pediatric Dialysis Emergency Machine) in five (2.0%) children. In 33 children, the device was reported as “other” and not specified, and in eight patients, it was not reported. The CVVHDF modality was used in the majority of patients (n = 175, 70.9%), regardless of the applied anticoagulation (RCA – 72.6%, HA – 64.3%, NA – 82.4%). The distribution of CKRT modalities did not differ regarding anticoagulation (Table 2). Small filters were utilised in 83 patients, medium in 94 patients, and large in 60 patients. All procedures were conducted via double-lumen central venous dialysis catheters (CVC). The median CVC diameter was 8.0 French (IQR 6.0–10.0) and was equal in all anticoagulation modalities (p = 0.253). The most common vascular access site was the right internal jugular vein (n = 116, 47.0%), followed by the femoral vein (n = 74, 30.0%), left internal jugular vein (n = 34, 13.8%), and subclavian vein (n = 21, 8.5%). The most common anticoagulation modality was HA (n = 112, 45.3%), followed by NA (n = 68, 27.5%) and RCA (n = 62, 25.1%). The median prescribed dialysis dose in the whole group was 2.4 L/h/1.73 m^2^ (IQR 1.73–3.46) or 53.0 mL/h/kg (IQR 36.1–85.7). The prescribed parameters of CKRT by anticoagulation modality are shown in Table 2. Blood flow rate differed between the anticoagulation groups (p = 0.003) and was lower in the RCA group than in the NA group (p = 0.002 post hoc analysis), whereas other dialysis parameters did not differ.

**Table 2:** Technical CKRT settings in the examined population

Parameter	HA (n = 112)	RCA (n = 62)	NA (n = 68)	P value
CKRT modality (n,%)				
CVVH CVVHD CVVHDF	26 (23.2%) 14 (12.5%) 72 (64.3%)	12 (19.3%) 5 (8.1%) 45 (72.6%)	9 (13.2%) 3 (4.4%) 56 (82.4%)	0.123
Blood flow rate [ml/min/kg]	4.05 (2.6–6.0)	3.1 (2.4–4.8)	5.0 (3.8–6.7)	0.003^1^
Dialysate flow rate [ml/h/kg]	27.7 (8.2–46.1)	25.0 (11.4–48.0)	26.9 (22.2–42.0)	0.776
Replacement fluid flow rate [ml/h/kg]	25.0 (10.3–46.7)	23.5 (15.1–46.7)	21.5 (14.8–34.1)	0.463
Dialysis dose [ml/h/kg]	55.5 (39.3–86.7)	52.4 (33.3–87.5)	47.7 (37.0–84.4)	0.595
Ultrafiltration [ml/h/kg]	2.6 (1.4–4.9)	1.6 (0.0–4.4)	3.4 (1.4–5.0)	0.077

Chi-squared test, Kruskal–Wallis ANOVA test

^1^post-hoc analysis – statistically significant difference between RCA and NA (p = 0.002)

No data on anticoagulation in 5 patients; incomplete data in 10 patients

HA, heparin anticoagulation; RCA, regional citrate anticoagulation; NA, no anticoagulation

### Circuit lifetime

**Fig. 3:**
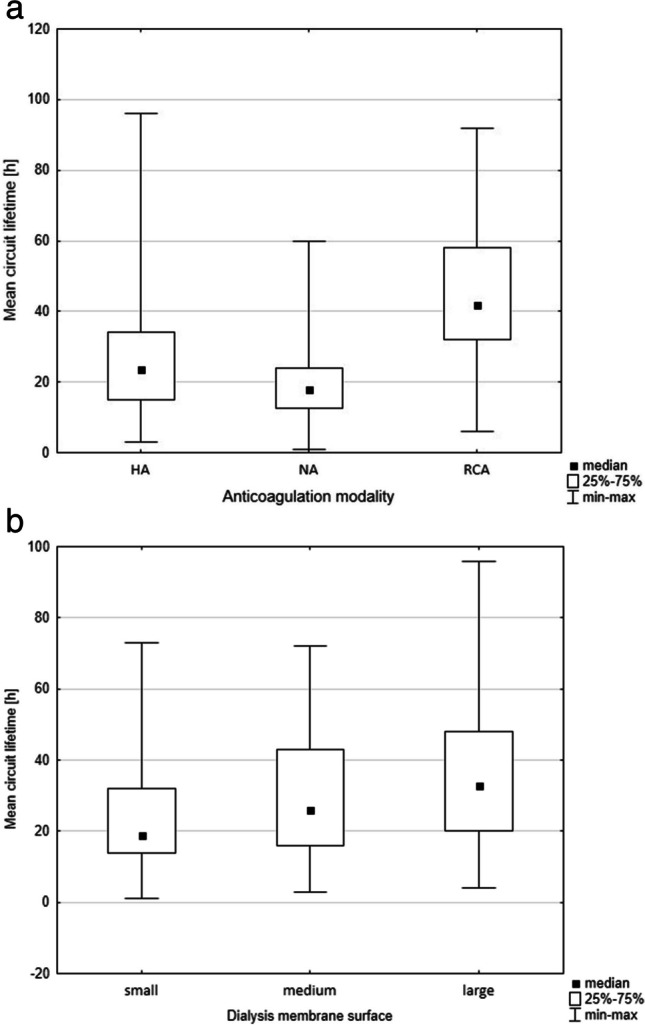
Mean circuit lifetime in the examined population according to anticoagulation modality (**a**) and dialyzer size (**b**). HA, heparin anticoagulation; RCA, regional citrate anticoagulation; NA, no anticoagulation

Independent of the anticoagulation, the median circuit lifetime in the entire group was 24 h (IQR 16.0–40.0). It was highest when RCA was used (42 h, IQR 32-58 h), lower with HA (24 h, IQR 14–34) and lowest when no anticoagulation was used (18 h, IQR 12–24), p < 0.001 (Fig. 3a). Circuit lifetime was longer with RCA than with HA and NA, independent of filter size (Table 3). In addition, filter size impacted mean circuit lifetimes (p < 0.001) (Fig. 3b). Small dialysers had shorter lifetimes (19 h, IQR 14–32) than both medium (26 h, IQR 16–43), (p = 0.044) and large ones (33 h, IQR 20–48), (p < 0.001) (Fig. 3b). In contrast, mean circuit lifetimes did not vary with the CKRT modality (p = 0.081).

**Table 3:** Mean circuit lifetime (in hours) in the examined population according to anticoagulation modality and the size of dialyzer membrane

	Entire cohort	HA n = 112	RCA n = 62	NA n = 68	P value
Entire cohort	-	23.5 (15.0–34.0)	42.0 (32.0–58.0)	18.0 (12.5–24.0)	< 0.001^1^
Small membrane n = 83	19.0 (14.0–32.0)	19.0 (13.0–32.0)	36.0 (28.5–38.5)	17.0 (13.0–22.0)	< 0.001^2^
Medium membrane n = 94	26.0 (16.0–43.0)	24.0 (12.0–36.0)	46.0 (31.0–62.5)	18.5 (13.0–39.0)	< 0.001^2^
Large membrane n = 60	33.0 (20.0–48.0)	30.0 (20.0–37.0)	48.0 (35.0–58.0)	19.0 (9.0–24.0)	< 0.001^2^
P value	< 0.001^3^	0.060	0.147	0.369	-

Kruskal–Wallis ANOVA test

^1^post-hoc analysis – difference between all 3 groups

^2^post-hoc analysis – statistically significant difference between RCA and HA, and between RCA and NA

^3^post-hoc analysis—difference between small and large membranes (p < 0.001)

HA, heparin anticoagulation; RCA, regional citrate anticoagulation; NA, no anticoagulation

Small membrane < 0.5m^2^, medium membrane 0.5-1m^2^, large membrane ≥ 1m^2^

No data on anticoagulation in 5 patients; no data on dialyzer membrane size in 10 patients; incomplete data in 10 patients

Circuit lifetimes were significantly longer in adolescents than in young children. However, this difference was lost when we divided the group by anticoagulation modality. Moreover, in all age groups except for children aged 6–12 years, the mean circuit lifetime was the longest with RCA, with no difference between HA and NA (Table 4). Anticoagulation modality distribution did not differ between the age groups. However, in adolescents, the RCA ratio was relatively higher than in other groups (Table 5).

**Table 4:** Mean circuit lifetime (in hours) in the examined population regarding the anticoagulation modality and patient age

	Neonates & infants	Young children	Children	Adolescents	p value
Mean circuit lifetime [h]	24.0 (17.0–37.0)	20.0 (15.0–38.0)	21.5 (12.5–47.5)	32.5 (24.0–45.0)	0.004^1^
HA [h]	26.5 (17.5–39.5)	20.0 (12.0–33.0)	15.0 (8.0–45.0)	29.0 (24.0–34.0)	0.076
RCA [h]	34.5 (28.0–40.0)	41.5 (31.0–60.5)	42.0 (21.0–71.0)	48.5 (40.0–59.0)	0.250
NA [h]	18.0 (14.0–24.0	16.5 (8.0–18.5)	22.0 (12.0–39.0)	22.0 (12.0–26.0)	0.369
p value	0.009	< 0.001	0.103	< 0.001	
post-hoc analysis					
RCA vs. HA	0.253	< 0.001	-	0.002	
RCA vs. NA	0.007	< 0.001	-	< 0.001	
HA vs. NA	0.228	0.257	-	0.481	

Statistical analysis: Kruskal–Wallis ANOVA test

^1^post-hoc analysis – difference between teenagers and young children (p = 0.002)

HA, heparin anticoagulation; RCA, regional citrate anticoagulation; NA, no anticoagulation

No data on anticoagulation in 5 patients; incomplete data in 10 patients

**Table 5:** Distribution of anticoagulation modalities and the occurrence of complications regarding the patient’s age

	Neonates & infants	Young children	Children	Adolescents	p value
Anticoagulation modality					
HA [n,%]	26 (46.4%)	42 (50.0%)	17 (43.6%)	27 (42.9%)	0.311
RCA [n,%]	12 (21.4%)	16 (19.1%)	11 (28.2%)	23 (36.5%)	
NA [n,%]	18 (32.2%)	26 (30.9%)	11 (28.2%)	13 (20.6%)	
Occurrence of dialysis-related complications [%]	35.1%	37.9%	30.8%	32.8%	0.857
HA	46.2%	38.1%	17.7%	37.0%	0.297
RCA	16.7%	6.3%	18.2%	13.0%	0.788
NA	33.3%	61.5%	63.6%	61.5%	0.221
p value	0.204	0.017	0.021	0.011	

Statistical analysis: chi-squared test

HA, heparin anticoagulation; RCA, regional citrate anticoagulation; NA, no anticoagulation

No data on anticoagulation in 5 patients; incomplete data in 10 patients

In univariate analysis, circuit lifetime correlated with BSA and dialysis membrane surface, but not with CVC diameter, blood, and dialysis fluid flow rates. In the centre-adjusted multivariate analysis, using the general step-wise regression model, the mean circuit lifetime was associated with dialyser surface area (beta = 0.14, 95% CI 1.3–12.6, p = 0.016), left internal jugular vein vascular access site (beta = -0.37, 95% CI -17.9 to -1.1, p = 0.027) vs. right internal jugular vein, and the use of HA (beta = -0.14, 95% CI -6.1 to -0.2, p = 0.038) or NA (beta = -0.37, 95% CI -12.3 to -5.9, p < 0.001) vs. RCA.

### Elective circuit changes

A total of 441 (32.5%) circuits were exchanged electively when the maximal suggested circuit time was reached. In the RCA group, the ratio of electively changed circuits (n = 107, 39.3%) was significantly higher than in other anticoagulation modalities (HA n = 192, 35.1%; NA n = 131, 24.9%; chi-squared test, p < 0.001). The remaining 11 electively changed filters were reported in 2 patients, with no anticoagulation data provided.

### Dialysis-related complications

Dialysis-related complications occurred in 86 patients (34.8%) and were related to the type of anticoagulation (p < 0.001). The lowest incidence was observed with RCA (12.9%), followed by HA (36.5%) and NA (54.4%). The incidence of complications was the lowest for RCA in young children (age 1–6 years) and adolescents. Catheter dislocation was the most common complication (37.2%). In univariate analysis, we found correlations between the occurrence of catheter dislocation and CVC diameter, as well as the femoral and left internal jugular vein vascular access site vs. the right internal jugular vein. However, in the centre-adjusted multivariate analysis, catheter dislocation was not affected by the vascular access site or the CVC diameter.

Anticoagulation-related complications (thrombosis/bleeding) occurred in 24 patients (27.9%). Thrombosis was reported in 16 patients (18.6% of all complications), with no significant difference between anticoagulation modalities (p = 0.356). Bleeding occurred in 8 of the patients (9.3% of all complications) who were undergoing CKRT with HA (n = 5) and NA (n = 3), respectively. No cases of bleeding were reported in patients on RCA-CKRT. In NA, the haemorrhagic complications were probably caused by coagulation defects related to the primary disease (liver disease, haematologic disease, and malignancy). In 30 patients the occurrence of complications was reported but not specified.

## Discussion

Paediatric CKRT presents a considerable number of challenges due to a wide variety of patient anthropometric characteristics, resulting in the necessity to use different-sized membranes and sets when compared to the adult population. Over the last years, CKRT has evolved into the leading dialysis technique in the paediatric intensive care setting [11]. Therefore, it is crucial to seek solutions to optimise the efficacy of paediatric CKRT procedures, which can be achieved, for example, by providing the maximum possible circuit longevity. In this study, we identified several independent factors affecting the circuit lifetime.

RCA superiority over HA in maintaining circuit survival was proven in an adult randomised control trial [9]. In the paediatric population, there is still no consensus regarding anticoagulation modality. Access to different anticoagulants varies worldwide. In our cohort, at least 77.7% of patients underwent CKRT on a device that includes the RCA software. In a recent European survey [12], RCA was the first-choice anticoagulation in 35% of centres. A global international study [13], as well as a worldwide systematic review [14], reported citrate as the most commonly used anticoagulant. Nafamostat mesylate [15] and prostacyclin [16] have also been applied with satisfactory results. Our current study, one of the largest paediatric CKRT cohorts to date, demonstrates clear advantages of RCA compared to HA and NA. RCA resulted in longer circuit lifetime independent of filter sizes and in different age groups, with no significant difference between HA and NA. This finding aligns with previous smaller observational studies [17–20], and a small crossover trial [21]. In contrast, a relatively large study from Australia [22] did not show a difference in circuit lifetime between RCA and HA, however, the results could have been influenced by the inclusion of patients on ECMO-related CKRT. A recent meta-analysis [23] and the latest systematic literature review [24] confirmed the superiority of RCA over HA in children. In addition, in our cohort, RCA showed the lowest incidence of dialysis-related complications thanks to the complete absence of bleeding complications. Our findings are in keeping with randomised controlled trial results in adults, where HA was associated with a higher risk of haemorrhage [25] and with higher transfusion rates [26] compared to RCA. It is of note that in our cohort, HA, while fraught with bleeding complications, did not prevent thrombotic complications.

The mean circuit lifetime was higher in adolescents than in children below the age of 6. This difference may be related to the use of larger circuits, as in our study, the dialyser membrane surface correlated positively with longer circuit survival. However, the difference between the age groups disappeared when we considered each anticoagulation modality separately. This discrepancy might be related to the relatively higher use of RCA in the group of adolescents. We also observed that RCA (compared to HA and NA) was associated with the lowest incidence of CKRT complications in all age groups. A study by Raymakers-Jansen et al. [20] showed that RCA significantly prolonged the circuit lifetime in children < 15 kg. Thus, RCA might be safe and effective even in the youngest children.

Another factor influencing the circuit lifespan identified in the present study is the filter membrane surface, with larger filters being associated with longer circuit survival in both uni- and multivariate analyses. The use of membranes below 0.5 m^2^ was associated with a shorter lifetime, which is consistent with a study by Cortina et al. [22]. Similarly, a study comparing HA and NA (but not RCA) [27] showed that filters larger than 0.4 m^2^ had higher longevity. Interestingly, in the present study, membrane size did not affect the mean circuit lifetime when RCA or NA was applied. A study by Miklaszewska et al. [28] including eight patients treated with RCA showed that the filter size influenced the circuit lifespan with no impact on anticoagulation modality. In our present analysis, we discovered that RCA is associated with significantly longer filter maintenance than both HA and NA, regardless of the filter size.

The last circuit lifetime predictor identified in the multifactorial analysis was the vascular access site. In line with clinical practice recommendations, the internal jugular vein was the most common vascular access site. However, our analysis showed that the left internal jugular vein access was associated with worse circuit longevity compared to the right site. Literature on this aspect is inconsistent. Data from the ppCRRT registry suggested superior circuit survival with internal jugular access [29]. Another study in a paediatric cohort showed no influence of the vascular access site on circuit longevity [22]. However, a meta-analysis of adult studies [30] showed a trend toward better filter survival with the femoral site compared to both jugular and subclavian accesses. Due to the divergent reported results, this aspect requires further research in the paediatric population before the optimal vascular CKRT access in children is established.

Interestingly, in our study, CKRT modality, dialysis dose, and blood flow did not correlate with circuit lifetime. The currently available data are inconsistent for all these points, and further research is required, especially in the paediatric population. Some studies reported that CVVHD [31], or CVVHDF [32, 33] may increase filter longevity, compared to CVVH. Higher blood flow rates may prolong circuit longevity by reducing clotting [34], and a recent meta-analysis [30] indeed suggested better circuit survival with higher blood flow rates. However, several paediatric observational studies showed no association of blood flow rate with circuit longevity [27, 35]. Interestingly, in our cohort, the blood flow rate was lower in patients receiving RCA. Hence, the lack of blood flow correlation with circuit lifetime may be partially related to the better efficacy of RCA even with low blood flow compared to the other modalities.

While our study represents one of the largest analyses of factors affecting CKRT function in children, it has several limitations. The multicentre character of the study limited our access to data that might have been useful but was not included in the EurAKId registry. Our study does not involve other anticoagulation modalities, such as nafamostat mesylate (currently unavailable in Europe) or prostacyclin. A high proportion of patients included in the registry come from the leading centre that initially designed the registry (see Supplementary Fig. 1). The mentioned leading centre has developed a regional protocol for RCA [11], which was described as safe and effective. This can cause bias, the risk of which we reduced by adjusting the multivariate analysis for centre, but also leads us to the conclusion that the development of unified protocols could improve the efficacy of CKRT in children. Furthermore, lack of data on individual circuit function precluded time-to-event analyses.

## Conclusions

Our prospective, multicentre, international registry study identified several factors influencing circuit lifetime in paediatric CKRT, in particular, anticoagulation modality, vascular access site, and dialyzer surface area. The present study demonstrates the superiority of RCA in maintaining CKRT functionality regardless of patient age, dialyser size, and blood flow rate. Hence, we conclude that RCA should be considered when choosing first-line anticoagulation for CKRT in children. Furthermore, the present study provides new data on aspects that are not yet clear and deserve to be extensively discussed to improve circuit survival and CKRT treatment in general. Further efforts should focus on developing guidelines and clinical practice recommendations for paediatric CKRT.

## Supplementary Information

Below is the link to the electronic supplementary material.

Graphical abstract (PPTX 160 KB)

Supplementary file2 (DOCX 26 KB)

## Data Availability

The datasets generated and analysed during the current study are available from the corresponding author on reasonable request.
