# Supplementary material for: Factors influencing circuit lifetime in paediatric continuous kidney replacement therapies – results from the EurAKId registry
Source: Pediatr Nephrol. 2024 Jul 18;39(11):3353–62. doi: 10.1007/s00467-024-06459-6 (PMC11413113; doi:10.1007/s00467-024-06459-6)
Supplement: Supplementary file 1 — Graphical abstract (PPTX 160 KB) [file 467_2024_6459_MOESM1_ESM.pptx]

## Slide 1
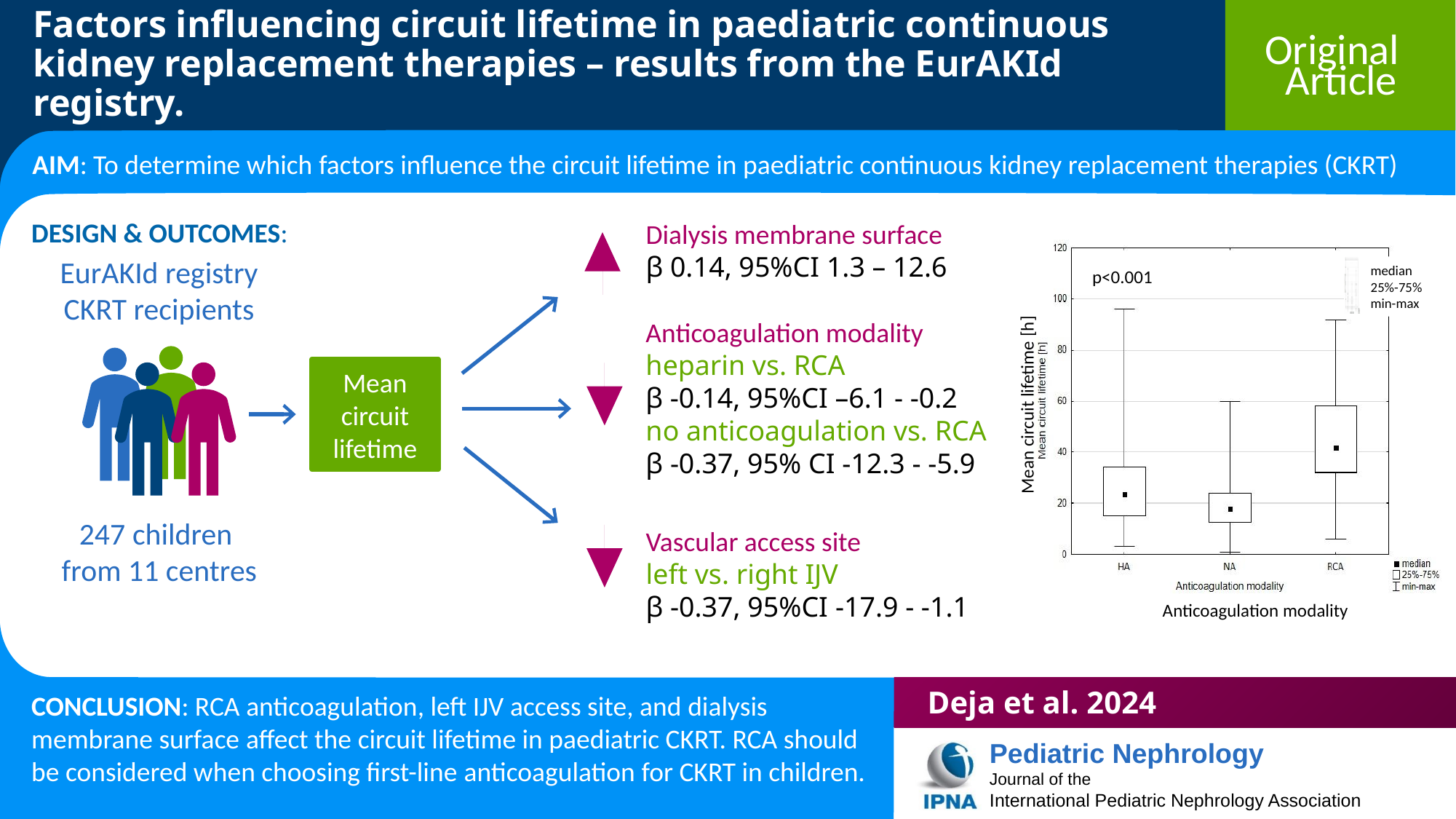

Factors influencing circuit lifetime in paediatric continuous kidney replacement therapies – results from the EurAKId registry.
AIM: To determine which factors influence the circuit lifetime in paediatric continuous kidney replacement therapies (CKRT)
DESIGN & OUTCOMES:
Dialysis membrane surface
β 0.14, 95%CI 1.3 – 12.6
p<0.001
Mean circuit lifetime [h]
Anticoagulation modality
median
25%-75%
min-max
EurAKId registry
CKRT recipients
Anticoagulation modality
heparin vs. RCA
β -0.14, 95%CI –6.1 - -0.2
no anticoagulation vs. RCA
β -0.37, 95% CI -12.3 - -5.9
Mean circuit lifetime
247 children
from 11 centres
Vascular access site
left vs. right IJV
β -0.37, 95%CI -17.9 - -1.1
Deja et al. 2024
CONCLUSION: RCA anticoagulation, left IJV access site, and dialysis membrane surface affect the circuit lifetime in paediatric CKRT. RCA should be considered when choosing first-line anticoagulation for CKRT in children.
