# Supplementary material for: Factors influencing circuit lifetime in paediatric continuous kidney replacement therapies – results from the EurAKId registry
Source: Pediatr Nephrol. 2024 Jul 18;39(11):3353–62. doi: 10.1007/s00467-024-06459-6 (PMC11413113; doi:10.1007/s00467-024-06459-6)
Supplement: Supplementary file 2 — Supplementary file2 (DOCX 26 KB) [file 467_2024_6459_MOESM2_ESM.docx]

| parameter | r | p |
| --- | --- | --- |
| CVC diameter | 0.13 | 0.052 |
| BSA | 0.18 | 0.007 |
| Dialysis membrane surface | 0.25 | <0.001 |
| Blood flow | -0.11 | 0.106 |
| Replacement flow/weight | 0.02 | 0.719 |
| Dialysate flow/weight | -0.01 | 0.853 |
| Dialysis dose/weight | -0.08 | 0.260 |
| Ultrafiltration/weight | -0.10 | 0.144 |

**Supplementary Table 1** Univariate correlations of the mean circuit lifetime with selected continuous variables

CVC – central venous catheter, BSA – body surface area

Spearman’s rank correlations

| parameter | beta | 95% confidence interval | p |
| --- | --- | --- | --- |
| Dialysis membrane surface | 0.14 | 1.3 – 12.6 | 0.016 |
| Vascular access site  (vs right internal jugular vein) |  |  |  |
| Subclavian vein | -0.16 | -15.4 – 5.9 | 0.382 |
| Left internal jugular vein | 0.17 | -17.9 - -1.1 | 0.027 |
| Femoral vein | -0.23 | -12.4 – 2.8 | 0.217 |
| Anticoagulation modality  (vs RCA) |  |  |  |
| HA | -0.14 | -6.1 – -0.2 | 0.038 |
| NA | -0.37 | -12.3 - -5.9 | <0.001 |

**Supplementary Table 2** Predictors of the mean circuit lifetime - multivariate analysis

General linear step-wise regression model

Adjusted for centres reporting the patients

**Supplementary Figure 1** Number of patients included in the study per centre

**Supplementary Figure 2** Primary disease occurrence in patients requiring CKRT for reasons other than AKI

no data on primary disease - 4 patients; no data on AKI occurrence -14 patients.
